# Retrograde Percutaneous Coronary Intervention in a Case of Takayasu Arteritis with Left Main Coronary Artery Chronic Total Occlusion

**DOI:** 10.1155/2022/3786613

**Published:** 2022-03-12

**Authors:** Prathap Kumar, Blessvin Jino, Stalin Roy, Manu Rajendran

**Affiliations:** Department of Cardiology, Meditrina Hospital, Kollam, Kerala, India

## Abstract

**Background:**

Takayasu arteritis (TA) frequently involves the coronary arteries, and restenosis is common after initial percutaneous coronary intervention (PCI). However, PCI remains a good option for patients who develop graft failure after coronary artery bypass graft surgery (CABG). Drug-coated balloons help in repeat revascularization after stent failure in TA. *Case Presentation*. A 31-year-old female with previous history of Takayasu arteritis (TA) and CABG with left internal mammary artery (LIMA) to left anterior descending artery (LAD) grafting in 2012, presented to us with exertional angina of 6-month duration. Her coronary angiogram showed total occlusion of the left main coronary artery (LMCA) ostium and atretic LIMA graft. Since the guiding catheter could not engage the LMCA, a retrograde approach was planned. After lesion crossing, the retrograde guidewire could not be externalized even after multiple attempts. Hence, a repeat antegrade approach was tried, and antegrade wire crossed through the channel created by the retrograde microcatheter. Then, intravascular ultrasound (IVUS) guided LMCA-LAD stenting was done. The patient was started on dual antiplatelets and prednisolone and was on regular follow-up. Three months later, the patient presented to us with non-ST elevation MI. Coronary angiogram showed critical in-stent restenosis of the LMCA stent, and optical coherence tomography (OCT) showed diffuse neointimal hyperplasia. OCT-guided PCI using sirolimus-coated balloon was done. On 8 months of follow-up, the patient remains symptom free.

**Conclusion:**

Coronary artery disease in TA may require repeated interventions due to stent/graft failure. Drug-coated balloons play a crucial role in repeat revascularization for stent failure in TA. Retrograde approach increases the technical success rate of PCI in LMCA-CTO.

## 1. Introduction

Coronary arterial lesions are seen in 53.2% of patients with TA on computed tomographic angiography [1]. Immunosuppressive therapies remain the primary treatment of organ ischemia caused by active disease, and interventions are recommended only in progressive organ ischemia [2]. We report an interesting case of left main coronary artery (LMCA) chronic total occlusion (CTO) which was managed by image-guided PCI.

## 2. Case Report

**Figure 1:**
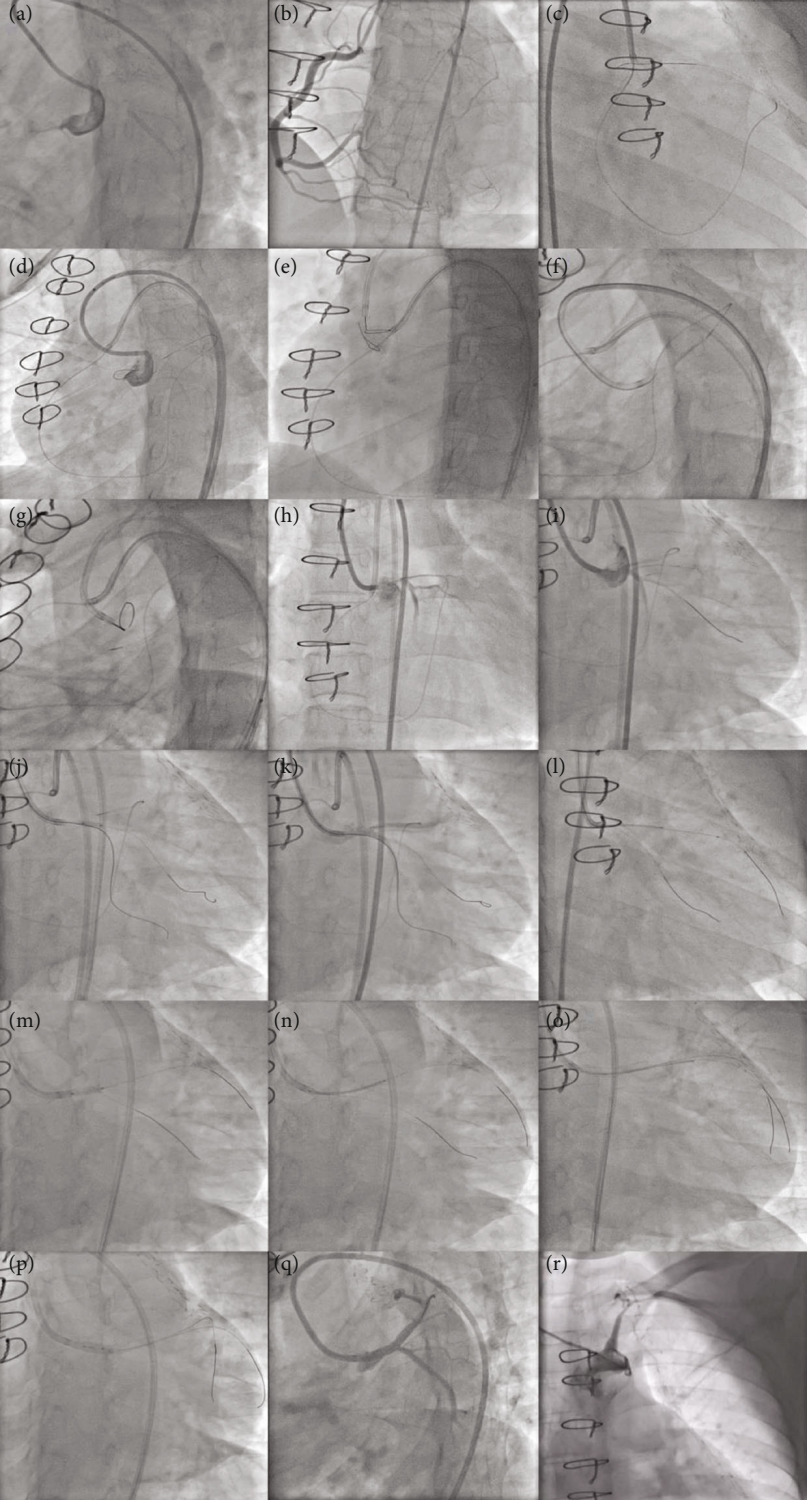
Angiographic images showing initial PCI for LMCA CTO. Left anterior oblique (LAO) caudal view showed LMCA total occlusion (a), and antero posterior (AP) cranial view showed normal RCA giving multiple collaterals to both LAD and LCX (b). LMCA lesion was crossed retrogradely using Fielder FC and caravel through septal collateral from RCA (c), and externalization into 7F EBU 3.5 antegrade guiding catheter was attempted by conventional method (d). After multiple failed attempts to enter antegrade guiding catheter, snaring was attempted using Amplatz goose neck snare (e). After multiple failed attempts of externalization and antegrade wiring (f–h), finally, SION black guidewire crossed LMCA lesion antegradely (i). Then, Fielder XTR guidewire was parked in LCX, and LMCA lesion was dilated with 2.5 × 8 mm NC balloon (j, k). After IVUS analysis (l), LMCA lesion was again dilated with 3 × 10 mm cutting balloon (m). Then, LMCA-LAD stenting was done with 3.5 × 26 mm ZES (n). After stenting, IVUS analysis showed underexpansion (o), and hence, postdilatation was done using 4 × 8 mm NC balloon (p). Final angiogram showed well expanded stent with good distal flow in LAD and LCX (q). Left subclavian artery angiogram showed stenosis at the proximal segment and atretic LIMA (r).

**Figure 2:**
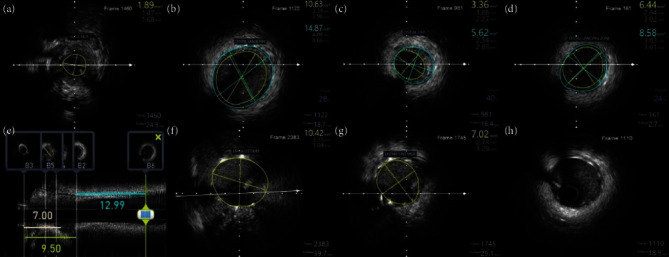
IVUS guidance in initial PCI optimization. Initial IVUS run showed LMCA MLA of 1.89 mm^2^ (a), and the mean distal LMCA vessel diameter was 4.36 mm (b). MLA of ostial LAD, mean vessel diameter of the distal landing zone, and the total lesion length were 3.36 mm^2^, 3.32 mm, and 22.5 mm, respectively (c–e). Post IVUS run showed ostial LMCA MSA of 10.42 mm^2^ (f) and ostial LAD MSA of 7.2 mm^2^ (g). The distal stent edge was free of edge dissection or malapposition (h).

**Figure 3:**
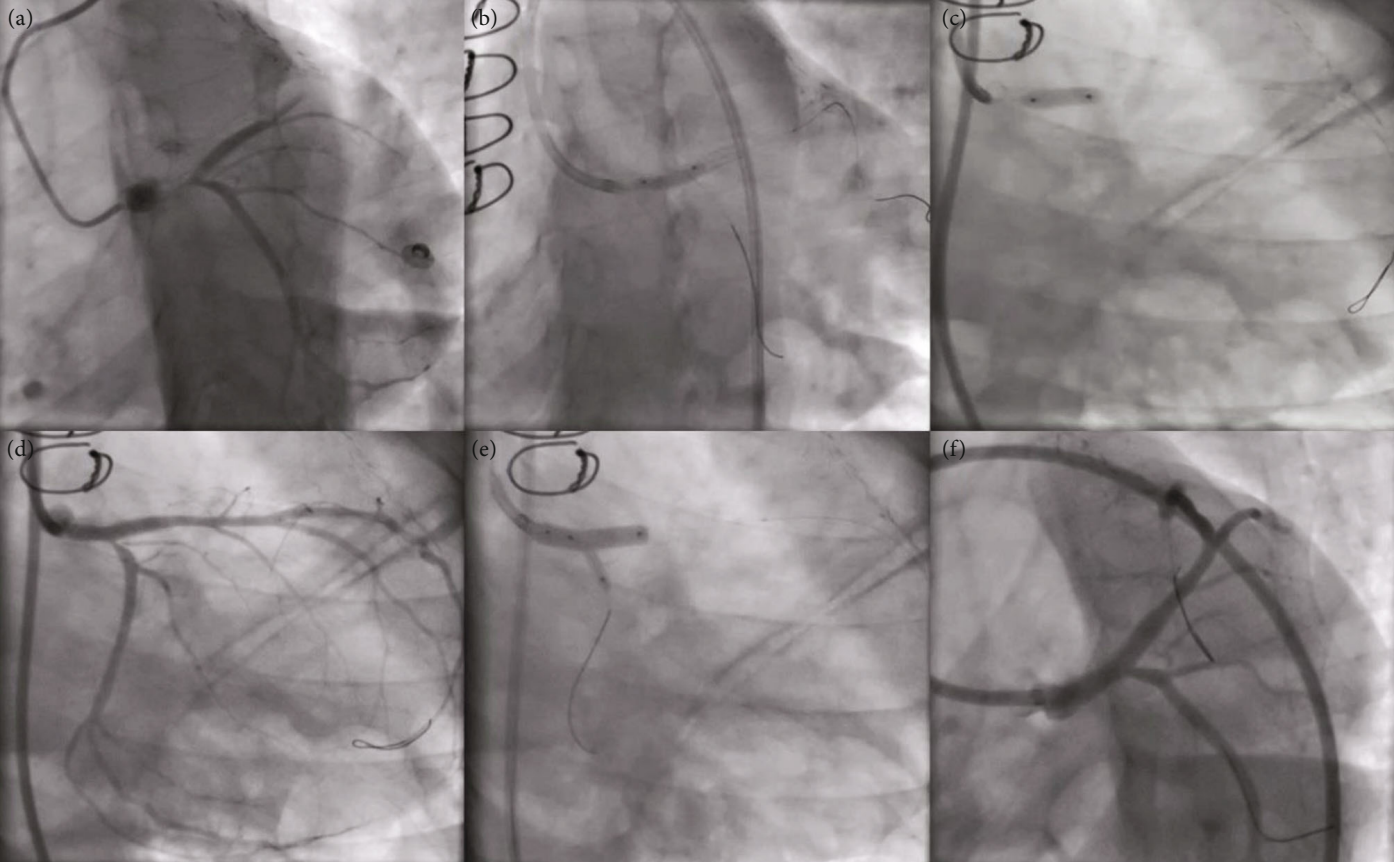
Angiographic images of repeat PCI. AP caudal view showed 95% ISR at proximal LMCA (a). Balloon dilatation of the lesion was done using 2.75 × 12 mm NC balloon and 3 × 10 mm cutting balloon (b, c). LCX ostium had critical narrowing after balloon dilatation (d), and hence, kissing balloon inflation (e) was done using 4 × 20 mm sirolimus eluting balloon (LMCA-LAD) and 2.75 × 12 mm NC balloon (LMCA-LCX). Final angiogram showed well expanded LMCA-LAD stent without residual stenosis (f).

**Figure 4:**
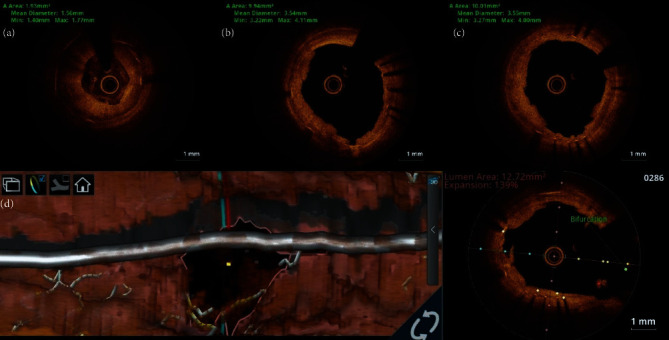
OCT images of PCI optimization. Initial OCT run showed diffuse neointimal hyperplasia with LMCA MSA of 1.93 m^2^ (a). Final OCT run showed MSA at proximal LMCA and ostial LAD as 9.94 mm^2^ and 10.01 mm^2^, respectively (b, c). OCT 3D bifurcation mode (ILUMIEN™ Optis™ System—Abbott Vascular) showed adequate LCX ostial area (d).

A 31-year-old female presented to our hospital with exertional angina class III of 6 months duration. She underwent coronary artery bypass grafting (CABG) with left internal mammary artery (LIMA) to left anterior descending artery (LAD) graft in 2012 for critical LMCA disease. She was diagnosed to have TA in 2012 and was on prednisolone 10 mg as maintenance therapy for TA. She had no other risk factors for coronary artery disease. Her electrocardiogram showed T inversion in the anterior leads, and echocardiogram showed left ventricular (LV) anterior wall hypokinesia with an ejection fraction (EF) of 50%. Her CRP level was 2 mg/dl on admission. Coronary angiogram showed LMCA ostial total occlusion and a normal right coronary artery giving collaterals to left system. The left subclavian artery had significant stenosis at the proximal segment, and the LIMA was atretic (Figure 1). The patient was planned for PCI to LMCA using primary retrograde approach through septal collaterals from RCA. RCA was engaged using 7F JR 3.5 guiding catheter, and septal collateral was crossed using Fielder FC guidewire and caravel. Since LMCA could not be engaged, 7F JL 3.5 guiding catheter was kept at the aortic root. After lesion crossing, guidewire could not enter the guiding catheter (Video 1), and hence, snaring was attempted using Amplatz goose neck™ snare and KAM snare (Videos 2 and 3). After multiple attempts of snaring, finally, antegrade wiring was tried through the channel created by the guidewire and caravel. SION black was used to cross the lesion antegradely and was parked in the left circumflex (LCX) artery. Then, the LMCA was dilated with a 2.5 × 8 mm noncompliant (NC) balloon, and then, Fielder XTR was parked at LAD. Again, after 3 × 12 mm NC balloon dilatation, intravascular ultrasound (IVUS) run was done. IVUS analysis showed that the disease involved both LMCA and proximal LAD (Video 4), and the minimal luminal area (MLA) of LMCA, mean LMCA vessel diameter, mean proximal LAD vessel diameter, and lesion length were 1.89 mm^2^, 4.36 mm, 3.32 mm, and 22.5 mm, respectively (Figure 2). In view of significant fibrotic lesion, another dilatation with 3 × 10 mm cutting balloon was done, and then, ostial LMCA to LAD stenting was done using 3.5 × 26 mm Zotarolimus eluting stent. Poststenting IVUS showed underexpansion at LMCA with stent area at ostial LMCA and ostial LAD were 10.42 mm^2^ and 7.02 mm^2^, respectively, without distal edge dissection or malapposition (Video 5). Finally, postdilatation was done with a 4 × 8 mm NC balloon, and the final angiogram showed a well expanded stent without any complications (Video 6). The patient was on regular medications including aspirin, ticagrelor, atorvastatin, and prednisolone and was on regular follow-up. Three months later, the patient presented with a non-ST elevation myocardial infarction, and she had COVID-19 vaccination 1 week prior to the chest pain. Her echocardiogram showed LV anterior wall hypokinesia with EF of 45%, and coronary angiogram showed LMCA shaft 95% in-stent restenosis (ISR) with distal TIMI-2 flow (Video 7, Figure 3). Ad hoc PCI was planned, and lesion was crossed with Fielder FC guidewires. After predilatation with a 2.75 × 12 mm NC balloon, optical coherence tomography (OCT) showed diffuse neointimal hyperplasia with LMCA MSA of 1.93 m^2^ (Video 8, Figure 4). Lesion was dilated with 3 × 10 mm Flextome™ and 4 × 20 mm sirolimus-coated balloon. Finally, kissing balloon inflation using 4 × 20 mm and 2.75 × 12 mm balloons and proximal optimization (POT) using 4.5 × 8 mm NC balloon were done. Final OCT run showed that stent areas at proximal LMCA, POC, and ostial LAD were 9.9 mm^2^, 8.4 mm^2^, and 10.01 mm^2^, respectively (Video 9). Final angiogram showed no residual stenosis without any complications (Video 10). The patient was discharged without any peri-procedural complications. On 8 months of follow-up, she remains symptom free.

## 3. Discussion

European League Against Rheumatism (EULAR) recommends vascular intervention in a patient with TA, when a vascular lesion persists despite medical therapy, particularly when it is associated with symptoms or risk of future complications [3]. Vascular interventions should be done preferably after controlling active inflammation, and interventions during active disease are associated with more complications [4] and low patency rates. Studies have shown that PCI is associated with higher risk of ISR and repeat revascularization comparing CABG in TA [5, 6]. In our case, the patient opted for PCI after detailed discussion with our heart team regarding the risks involved in redo CABG and the possibilities of ISR in PCI. LMCA-CTOs are seen mostly in post-CABG patients, and PCI for LMCA-CTO is performed only rarely, around 0.45% of all CTO PCIs in one registry [7]. PCI to an unprotected LMCA-CTO is technically challenging. Subintimal entry and balloon dilatation may compromise the flow in LAD or LCX and may become life-threatening. Hence, dissection reentry techniques are not advisable in an unprotected LMCA-CTO. Though an antegrade approach is possible in distal LMCA-CTOs, a retrograde approach is preferred in ostial LMCA-CTOs due to lack of guide catheter support for an antegrade approach. Externalization of guidewire by pushing the retrograde guidewire into an antegrade guide catheter is technically difficult in ostial LMCA-CTOs, since the guide catheter cannot be engaged, and it has to be floated in the aorta near the LMCA ostium. Hence, snaring may be a better option. PCI with drug-coated balloon (DCB) without stenting is another option in patients with recurrent restenosis, and it has reasonably good outcomes [8]. Intracoronary imaging is essential to delineate the underlying cause for the ISR, because treatment varies according to the underlying etiology [9]. Though both OCT and IVUS can be used in stent failure, OCT is the preferred option because of its excellent resolution to detect the underlying biological causes. Both DCB and drug eluting stents (DES) are recommended for ISR due to biological causes like neointimal hyperplasia and neo-atherosclerosis [10].

## 4. Conclusion

TA may present with complex coronary artery disease, and its treatment is associated with a high incidence of repeat revascularization. Intracoronary imaging helps in identifying the underlying mechanism for stent failure, which directs the further management. PCI using drug-coated balloons helps to avoid repeated stenting in patients with TA and stent failure. Retrograde approach increases the success rate of PCI in LMCA-CTOs, particularly in ostial LMCA-CTO.
